# Pain catastrophizing and associated factors in preoperative total knee arthroplasty in Lanzhou, China: a cross-sectional study

**DOI:** 10.1186/s12891-022-05435-1

**Published:** 2022-05-28

**Authors:** Juhong Pei, Haixia Chen, Tong Ma, Ying Zhang, Xiangfu Wang, Chenxu Li, Binglin Ye, Xingsheng Wang, Jirong Zhao, Xinman Dou

**Affiliations:** 1 The First Clinical Medical College, Lanzhou University, Lanzhou, Gansu China; 2 Oncology Surgery Department, Lanzhou University Second Hospital, Lanzhou, China; 3 Department of Spine Minimally Invasive Orthopedics, Gansu Provincial Hospital of Traditional Chinese Medicine, Lanzhou, Gansu China; 4 Department of Anesthesia and Surgery, Gansu Provincial Hospital of Traditional Chinese Medicine, Lanzhou, Gansu China; 5 Department of Emergency, Gansu Provincial Hospital of Traditional Chinese Medicine, Lanzhou, Gansu China; 6 Clinical college of Traditional Chinese Medicine, Gansu university of Chinese medicine, No. 418, guazhou Road, Qilihe District, Lanzhou, 730000 Gansu Province China; 7 The First Affiliated Hospital, Gansu University of Traditional Chinese Medicine, Lanzhou, Gansu, China; 8 Department of Nursing, Lanzhou University Second Hospital, Lanzhou, China

**Keywords:** Pain catastrophizing, Anxiety, Pain, Associated Factors, Total Knee Arthroplasty

## Abstract

**Background:**

Pain catastrophizing in preoperative total knee arthroplasty (TKA) patients is associated with several poorly characterised factors in the literature. This study investigated the current state and associated factors of preoperative pain catastrophizing in patients undergoing TKA.

**Methods:**

This descriptive cross-sectional study was conducted at the orthopedics ward of two tertiary hospitals in Lanzhou, China. Pain catastrophizing was measured using the Chinese versions of the Pain Catastrophizing Scale, Short Form-36 (physical function domain), Numerical Rating Scale, Oxford Knee Score, Hospital Anxiety and Depression Scale, and Life Orientation Test-Revised.

**Results:**

The study included 360 participants. Preoperative TKA pain catastrophizing in all patients was high, with a mean score of 24.92 (SD: 12.38). The stepwise multiple linear regression analysis revealed anxiety (*β* = 0.548, *P* < 0.01), education level (*β* =  − 0.179, *P* < 0.01), physical function (*β* =  − 0.156, *P* < 0.01), and pain intensity during activity (*β* = 0.105, *P* = 0.015) as associated factors for pain catastrophizing, possibly explaining 51.2% of the total variation (*F* = 95.149, *P* < 0.01).

**Conclusion:**

Anxiety was the most relevant factor for pain catastrophizing in patients with preoperative TKA. Lower education levels, poor physical function, and stronger pain intensity during the activity were also associated with pain catastrophizing.

## Introduction

Total knee arthroplasty (TKA) is a common surgical option in patients with end-stage knee osteoarthritis. It is more effective than non-surgical treatment for relieving pain, stiffness, and mobility restrictions, as well as improving life quality [1, 2]. TKA procedures have increased substantially in the past 20 years and are expected to grow worldwide as the obesity epidemic expands and the population ages [1, 3]. For example, between 1992 and 2011, the number of primary TKAs performed in the United States more than tripled, from 203,600 to 645,100 [4].

Although most patients undergoing primary TKA recuperate well, about 10%-34% of post-surgical patients experience persistent pain [5]. The persistent, chronic pain increases patients’ health burden, negatively affecting the quality of life and reducing the satisfaction of surgery [6]. Gender, level of education, comorbidities, body mass index, social support, or other surgical factors cannot fully explain this outcome [5, 7]. Growing evidence suggests the potential effect of preoperative psychological distress, such as pain catastrophizing, anxiety, depression, and poor coping skills, on the development of persistent pain after TKA [8–11], with the role of pain catastrophizing being increasingly considered.

Pain catastrophizing is characterized by excessive focus on pain symptoms (rumination), exaggerated rating of the threat value of pain (magnification), and awareness to be unable to control the pain (helplessness) [12, 13]. Catastrophizing in a pain context can reduce the patient’s compliance with the training program and may have a negative impact on the severity of pain after TKA [12]. Growing evidence demonstrates that preoperative pain catastrophizing has a negative impact on patients undergoing TKA, often leading to persistent pain and poor function [14, 15]. In a systematic review, Sorel et al. examined the effect of preoperative psychological distress on pain and function after TKA. The review demonstrated the negative impact preoperative pain catastrophizing has on pain and function [11]. Burns et al. provided moderate-level evidence for pain catastrophizing as an independent predictor of chronic pain persisting for ≥ 3 months after TKA [10]. Riddle et al. indicated that the risk of chronic postsurgical pain was more than twice in patients with high levels than in patients with low levels of pain catastrophizing [16]. Further, the results of a previous study demonstrated that the preoperative level of pain catastrophizing in patients determine, in combination with other variables, the length of an inter-individual variation in hospital stay after TKA [17].

To the best of our knowledge, studies exploring the related factors of pain catastrophizing in preoperative TKA in China are lacking. Therefore, this study investigated the current state and associated factors of preoperative pain catastrophizing in patients undergoing TKA in Lanzhou, China. The study results may provide evidence for further research on developing intervention strategies for patients with pain catastrophizing undergoing TKA.

## Materials and methods

### Study design

This descriptive cross-sectional study was conducted from July to December 2020 at the orthopedics ward of two tertiary hospitals in Lanzhou, China. The study was approved by the Medical Ethical Committee of Lanzhou University second hospital (Approval Number: 2020A-126).

Patients with a diagnosis of primary knee osteoarthritis, scheduled for primary unilateral TKA secondary knee osteoarthritis, having the ability to speak, write, and understand the Chinese language, aged at least 18 years, and willing to provide informed written consent were included in the study. Patients with prior knee surgery or scheduled for revision or unicondylar knee arthroplasty, cognitive and/or neurological disorders that prevented understanding of the questionnaires and surveys, or complications from other serious chronic diseases (e.g., cancer, heart failure, kidney failure) were excluded.

**Fig. 1:**
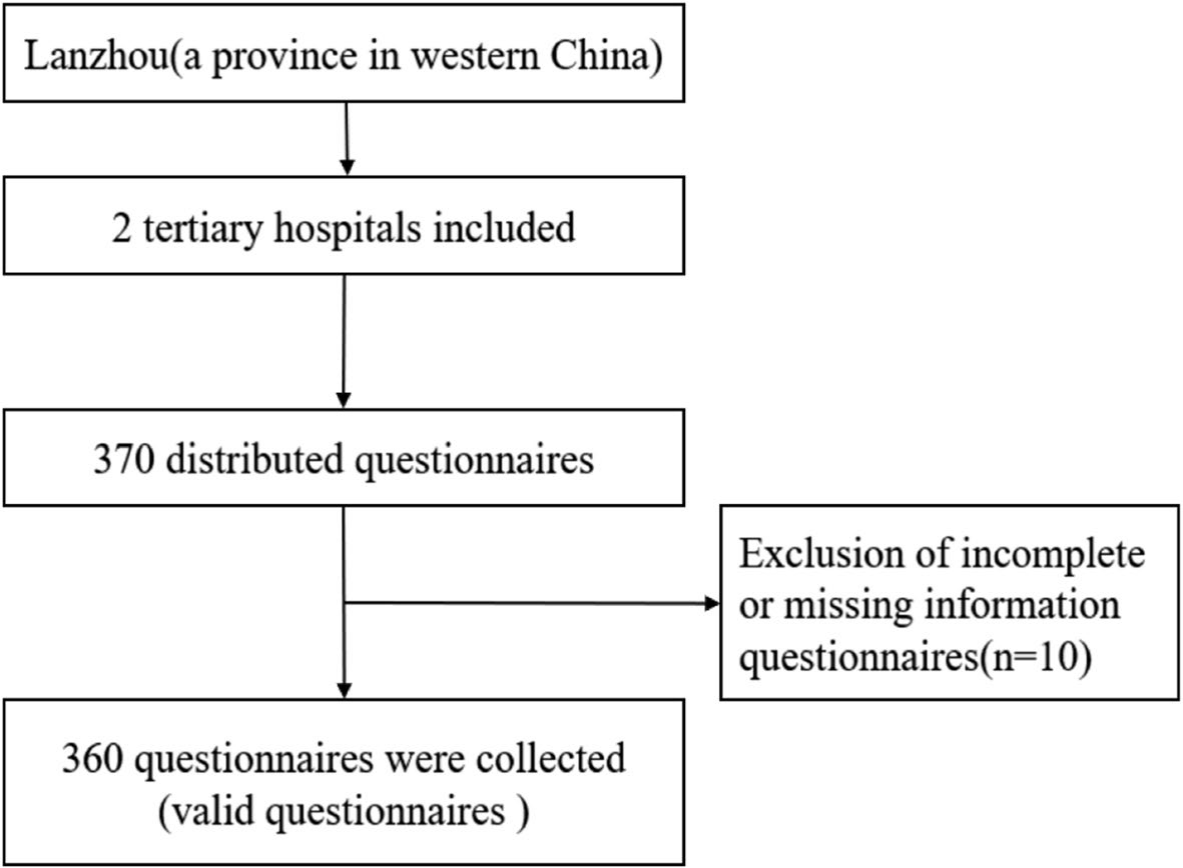
Flowchart of all eligible patients and reasons for non-participation

Following study enrollment, each patient completed six preoperative questionnaires, under the guidance of well-trained investigators, on the first day of their arrival at the hospital. Investigators participated in training sessions to ensure consistency in data collection. During these sessions, the investigators were asked to complete a questionnaire simultaneously. The evaluation results were checked for the discrepancy, and the investigators were trained on how to reach a consensus in case of disagreements. All data were filled independently by the patients within 30 min, and the investigators were present throughout the visit to provide explanation or clarification if needed. In addition, participants were provided unified verbal guidance regarding the questionnaires. Of the 370 structured questionnaires distributed, 10 were excluded because of incomplete or missing information (Fig. 1).

## Dependent measure

### Pain catastrophizing

Pain catastrophizing was measured by the Chinese version of the Pain Catastrophizing Scale (PCS) [18]. The scale includes 13 items that describe thoughts and feelings that patients may experience when in pain. The scale has three dimensions: rumination (4 items), magnification (3 items), and helplessness (6 items). Patients rate their recent pain-related thoughts using a 5-point Likert scale ranging from 0 (“not at all”) to 4 (“all the time”). The PCS total score is calculated by totaling the 13 items that vary between 0 (no catastrophizing) and 52 (severe catastrophizing), with a higher score indicating a higher perceived level of catastrophizing. Sullivan et al. proposed a PCS score of ≥ 30 as a cut-off point for pain catastrophizing [13]. This cutoff was used in previous studies, with ≥ 16 representing a high degree of pain catastrophizing [19, 20]. However, based on a Chinese study, we used a cutoff score of ≥ 38 to represent a high degree of pain catastrophizing [18]. In that Chinese study, the PCS was linguistically translated and culturally adapted, and the meaning of the original version was adequately retained by idiomatic translation. The Chinese version of the PCS has shown a high internal consistency, with a Cronbach’s alpha of 0.93 in patients with chronic pain.

## Independent measures

### Sociodemographic information

Sociodemographic information included data on age, gender, weight, height, marital status, educational level, address, medical insurance type, and work status.

### Physical function

The Short Form-36 (SF-36) is a widely used generic scale consisting of 36 items in 8 sections to evaluate 8 different domains. The participants’ physical function (PF) was assessed by the PF domain in the Chinese version of the SF-36 [19]. The PF domain comprises 10 items and is scored on a scale from 0 to 100, with higher scores indicating better PF.

### Pain intensity

Preoperative knee pain intensity was measured by a Numerical Rating Scale (NRS), with 0 indicating no pain and 10 the worst imaginable pain. These pain scores reflect the participant’s pain at rest or during activity.

### Oxford Knee Score

The Chinese version of the Oxford Knee Score (OKS) is a self-reported joint-specific questionnaire of pain and function associated with the knee [21]. The scale comprises 12 items on a 5-point Likert scale, with the total score ranging from 0 to 48 and lower scores indicating better functional status.

### Symptoms of anxiety and depression

The participants' individual anxiety and depressive symptoms were evaluated using the Hospital Anxiety and Depression Scale (HADS), which comprises two 7-item subscales, including anxiety and depression [22]. Response scores for the 14 items range from 0 to 3. The scores of the two subscales are calculated by the mean scores of the corresponding items for the scores ranging from 0 to 21. A score on a subscale is classified into three categories: within the normal range (0–7), suspected anxiety or depression (8–10), and presence of anxiety/depression (≥ 11). The HADS is reliable and valid in Chinese populations, with Cronbach alpha coefficients for the two subscales of 0.76 and 0.79 [23].

### Optimism and pessimism

Optimism and pessimism were assessed using the Chinese version of the Life Orientation Test-Revised (LOT-R) [24]. This scale consists of 10 items, which are divided into optimism (3 items), pessimism (3 items), and filler (4 items) items. Subjects respond to each item using a 5-point Likert score, ranging from 0 (not at all in agreement) to 4 (very much in agreement). The two subscale scores are calculated, and the total score adds the optimism score to the inverted pessimism score. The high and low scores represent bias towards the characteristics described by the dimension, that is, the higher the optimism score, the more optimistic the temperament is, and the higher the temperament pessimism score is, the more pessimistic it is.The LOT-R has satisfactory psychometric properties (Cronbach’s alpha = 0.78) to measure optimism and pessimism.

## Statistical methods

Statistical analyses were performed using the SPSS 25.0 (IBM Corp., Armonk, NY, United States). The Kolmogorov–Smirnov test was performed to examine whether the continuous variables were normally distributed. Count data are presented as frequency and percentage (%), and measurement data are described as means ± standard deviation (SD). Initially, independent two-samples *t*-test and ANOVA were used to compare the pain catastrophizing score for different demographic characteristics of patients. Then, Pearson correlation analysis was used to analyze the correlation between variables. Then, multivariate linear stepwise regression analysis was conducted with pain catastrophizing as a dependent variable and the following potential predictor variables that were revealed to be statistically significant in the univariate analyses as an independent variable: gender, education level, marital status, address, medical insurance type, working status, PF, OKS, pain intensity at rest, pain intensity during activity, anxiety, and depression. As high correlations among the predictor variables may lead to multicollinearity, the variance inflation factor (VIF) was inspected for evidence of multicollinearity in the model. A *P* value of < 0.05 was considered statistically significant for all analyses.

## Results

### Participant sociodemographic characteristics and their influence on pain catastrophizing

The total pain catastrophizing score of preoperative TKA patients was 24.92 ± 12.38. The scores of each dimension were as follows: rumination: 9.00 ± 4.04; magnification: 5.08 ± 2.91; and helplessness: 10.83 ± 6.31. In total, 360 patients were screened and 70 of them had a PCS score of ≥ 38. The descriptive statistics of the relevant factors of pain catastrophizing are presented in Table 1. Results of the ANOVA (Table 2) revealed a significant difference among preoperative TKA patients in the pain catastrophizing score with regards to gender (*F* =  − 2.412,* P* = 0.016), education level (*F* = 9.934,* P* < 0.01), marital status (*F* = 2.088, *P* = 0.038), address (*F* =  − 2.847, *P* = 0.005), medical insurance type (*F* = 8.869, *P* < 0.01), and working status (*F* =  − 3.734, *P* < 0.01).

**Table 1:** Descriptive statistic of the study variables (*N* = 360)

	Pain catastrophizing	Physical function	Pain intensity		Oxford Knee Score	Anxiety	Depressive	Optimism	Pessimism
			At rest	During activity					
Mean	24.92	37.18	3.66	7.26	27.56	7.50	6.66	8.27	6.45
SD	12.38	20.33	2.14	1.82	8.19	4.37	4.54	2.05	2.17
Range	0–52	0–95	0–10	1–10	3–48	0–21	0–21	0–12	0–12

**Table 2:** Participants’ characteristics and pain catastrophizing (*N* = 360)

	Variable	n(%)	Catastrophizing score	Statistic	
			Mean ± SD	*t/F*	*P*
Gender	Male	61 (16.9)	21.46 ± 13.40	-2.412	0.016
	Female	299 (83.1)	25.63 ± 12.11		
Age	< 60	63 (17.5)	26.54 ± 12.58	2.355	0.096
	60 ~	211 (58.6)	25.42 ± 12.21		
	≥ 70	86 (23.9)	22.51 ± 12.46		
Education level	Illiteracy	118 (32.8)	29.46 ± 11.33	9.934	< 0.01
	Primary school	122 (33.9)	23.81 ± 12.29		
	Middle school	54 (15.0)	23.43 ± 12.02		
	High school and above	66 (18.3)	20.08 ± 12.29		
Marital status	Married	305 (84.7)	25.50 ± 12.43	2.088	0.038
	Divorced or widowed	55 (15.3)	21.73 ± 11.70		
BMI(kg/m2)	< 18.5	8 (2.2)	31.00 ± 10.37	1.646	0.178
	18.5 ~	117 (32.5)	26.04 ± 13.22		
	24 ~	160 (44.4)	24.74 ± 11.50		
	≥ 28	75 (20.8)	22.91 ± 12.84		
Address	Rural area	216 (60.0)	26.42 ± 12.26	-2.847	0.005
	County town and Urban area	144 (40.0)	22.67 ± 12.56		
Medical insurance type	Own expense	5 (1.4)	27.00 ± 11.42	8.869	< 0.01
	SMI and URMI	133 (36.9)	21.41 ± 12.93		
	NRCMI	222 (61.7)	26.98 ± 12.24		
Working status	Yes	94 (26.1)	20.89 ± 11.623	-3.734	< 0.01
	No	266 (73.9)	26.34 ± 12.34		

ANOVA analysis was used to compare the mean score of pain catastrophizing among different groups of preoperative TKA patients

*SMI* Staff Medical Insurance, *URMI* Urban Residents Medical Insurance, *NRCMI* New Rural Cooperative Medical Insurance

### Relationships between the study variables

Pearson’s correlation analysis (Table 3) demonstrated that pain catastrophizing was significantly negatively correlated with PF (*r* =  − 0.416, *P* < 0.01) and significantly positively correlated with OKS (*r* = 0.516, *P* < 0.01), pain intensity at rest (*r* = 0.375, *P* < 0.01), pain intensity during activity (*r* = 0.407, *P* < 0.01), anxiety (*r* =  − 0.662, *P* < 0.01), and depression (*r* = 0.596, *P* < 0.01).

**Table 3:** Pearson correlation coefficients among the study variables (*N* = 360)

	1	2	3	4	5	6	7	8	9
Pain catastrophizing (1)	1	-0.416^**^	0.516^**^	0.375^**^	0.407^**^	0.662^**^	0.596^**^	0.028	-0.038
Physical function (2)		1	-0.629^**^	-.0360^**^	-0.442^**^	-0.359^**^	-0.401^**^	-0.009	-0.014
OKS (3)			1	0.532^**^	0.609^**^	0.575^**^	0.552^**^	0.001	0.033
Pain intensity at rest (4)				1	0.618^**^	0.408^**^	0.400^**^	-0.013	-0.054
Pain intensity during activity (5)					1	0.384^**^	0.399^**^	-0.060	-0.041
Anxiety (6)						1	0.833^**^	0.015	0.026
Depression (7)							1	0.048	0.129^*^
Otimism (8)								1	0.223^**^
Pessimism (9)								^*^	1

*OKS* Oxford Knee Score

^**^ *P* < 0.01, * *P* < 0.05 (two-tailed)

### Associated factors of preoperative pain catastrophizing among TKA patients

Collinearity analysis indicated absence of multicollinearity in all independent variables. Considering pain catastrophizing as the dependent variable, the statistically significant variables in Tables 2 and 3 were included as independent variables in the stepwise multiple linear regression equation for analysis. The model was statistically significant (*F* = 95.149, *P* < 0.01), explaining 51.2% of the total variance of pain catastrophizing. Anxiety (*β* = 0.548, *P* < 0.01), education level (*β* =  − 0.179, *P* < 0.01), PF (*β* =  − 0.156, *P* < 0.01), and pain intensity during activity (*β* = 0.105, *P* = 0.015) were the associated factors of pain catastrophizing (Table 4).

**Table 4:** Multiple linear regression analyses predicting pain catastrophizing (*N* = 360)

Dependent variable	Independent variable	*B*	*SE*	*β*	*t*	*P*
Pain catastrophizing	(Constant)	16.096	2.889		5.571	< 0.01
	Anxiety	1.553	0.116	0.548	13.357	< 0.01
	Education level	-2.039	0.425	-0.179	-4.802	< 0.01
	Physical function	-0.095	0.026	-0.156	-3.691	< 0.01
	Pain intensity during activity	0.713	0.291	0.105	2.451	0.015

*B* standardized Beta, *SE* Standard Error

*R* = 0.719*, R*^*2*^ = 0.517, Adjusted *R*^*2*^ = 0.512, *F* = 95.149, *P* < 0.01

## Discussion

The study investigated the current state of pain catastrophizing and its associated factors among preoperative TKA patients. Pain catastrophizing was higher (24.92 ± 12.38) in our study than in previous studies of preoperative TKA patients in Norway (18.2 ± 12.10) [25], the United States (12.0 ± 10.70) [20], and Germany (14.5 ± 8.3) [26]. In addition, we found that anxiety, education level, PF, and pain intensity during the activity were associated factors of pain catastrophizing. At the univariate level, we also found that pain catastrophizing was correlated with gender, marital status, address, medical insurance type, and working status.

Our results revealed that Chinese preoperative TKA patients had higher levels of pain catastrophizing, similar to Wang et al.’s research findings (2013) [27]. The literature has varied results of the levels of pain catastrophizing, which could be attributed to differences in sample size and sociodemographic characteristics of the participants. In addition, differences in regional and cultural backgrounds may also affect the results of the level of pain catastrophizing. In a previous study, African-Americans reported higher pain catastrophizing than white Americans [28]. In another study, Chinese undergraduates reported higher levels of pain catastrophizing than European Canadian undergraduates [29].

This study found that participants with lower education levels were more likely to experience pain catastrophizing than those with higher education levels. This finding was consistent with the results of a prior study demonstrating a correlation between lower education level and higher pain catastrophizing scores [20]. A study in patients with lumbar spinal stenosis also found that those with higher education levels presented with significantly lower pain catastrophizing scores than those with lesser education levels [30]. Previous studies demonstrated that education level determines the individual’s conceptualization of disease and their cognitive assessment of physical symptoms and that chronic pain patients with lower education levels are more likely to possess maladaptive pain beliefs and coping strategies [31, 32]. Another likely explanation for the present findings may be that individuals with lower education levels may be particularly disadvantaged in acquiring and assimilating medical knowledge by which to understand and address their pain concerns [33].

We found anxiety scores to be the most relevant variable for pain catastrophizing in preoperative TKA patients. This finding is similar to the results of a previous study indicating an association between anxiety and pain catastrophizing in individuals with chronic pain and anxiety as an important factor mediating the relationship between pain catastrophizing and prescription opioid misuse [34]. In addition, a recent study by Fillingham et al. [35] concluded that screening for preoperative anxiety and referral for treatment might improve patient outcomes and reduce opioid consumption following TKA. Furthermore, considering the higher incidence of anxiety in preoperative TKA patients [36], developing targeted interventions for anxiety may be crucial to improve pain catastrophizing. Therefore, healthcare professionals must consider anxiety when assessing pain catastrophizing in preoperative TKA patients.

We also found that PF was significantly associated with pain catastrophizing, consistent with a previous study finding that poor PF is linked to high pain catastrophizing [20]. Birch et al. [37] also found that patients with high levels of preoperative pain catastrophizing have lower PF. Similarly, in a study by Sullivan et al. [38], pain catastrophizing was found to predict both pain and function 12 months after TKA. The study by Bierke et al. [39] also showed that patients with high pain catastrophizing scores have a significantly lower total KOOS score preoperatively and 6 months postoperatively. On the contrary, patients with TKA have long-term knee osteoarthritis before surgery and chronic knee joint pain that is repetitive, progressing, and aggravated after movement, possibly leading to a feeling of helplessness in pain control. However, individuals with higher levels of pain catastrophizing are likely to engage in avoidance and fear of movement and physical activity [40, 41]. None of these studies investigated the causal relationship between pain catastrophizing and PF.

Similar to previous studies [42, 43], we found that patients with stronger pain intensity during the activity were more likely to experience pain catastrophizing. The result is consistent with the findings of a recent study by Larsen et al. [44], who pointed out that preoperative clinical pain intensity, high levels of pain catastrophizing thoughts, and impaired conditioned pain modulation may predict long-term postoperative pain 12 months after TKA. A review by Quartana et al. indicated [45] an association between catastrophizing and pain intensity. It is speculated that patients with stronger pain intensity may over-conceive and exaggerate the impact of pain on their own health and, thus, are more likely to develop pain catastrophizing.

## Study limitations

This study has certain limitations. First, the study had a cross-sectional design. As such, the causal relationship between pain catastrophizing and other variables in preoperative TKA patients cannot be explained. Second, the study was performed in two tertiary hospitals in China, and the results cannot be generalized to other groups. Third, only a few preoperative factors were examined, and only quantitative research methods were used, making it difficult to understand other factors that may also influence pain catastrophizing.

## Conclusion

In this study, we found that the level of pain catastrophizing is high in preoperative TKA patients. Anxiety, education level, PF, and pain intensity during activity were associated with pain catastrophizing. Because some of those factors are modifiable, it is necessary to consider them when formulating targeted interventions to manage pain catastrophizing in preoperative TKA patients.

## Data Availability

The datasets used and/or analysed during the current study are available from the corresponding author on reasonable request. Human subject protection requirements, appropriate data privacy as well as institutional requirements must be met.
